# Editorial: Long-read sequencing—Pitfalls, benefits and success stories

**DOI:** 10.3389/fgene.2022.1114542

**Published:** 2023-01-04

**Authors:** Inken Wohlers, Shilpa Garg, Jayne Y. Hehir-Kwa

**Affiliations:** ^1^ Medical Systems Biology, Lübeck Institute for Experimental Dermatology (LIED) and Institute for Cardiogenetics, University of Lübeck, Lübeck, Germany; ^2^ Department of Biology, University of Copenhagen, Copenhagen, Denmark; ^3^ NNF Center for Biosustainability, Technical University Denmark, Copenhagen, Denmark; ^4^ Princess Maxima Center for Pediatric Oncology, Utrecht, Netherlands

**Keywords:** sequencing, third-generation sequencing, long-read sequencing, nanopore, PacBio, genetic variants, genomics, transcriptomics

Long-read sequencing is an approach that holds promise to obtain genomic information for the first time in its entirety, accurately and resolved by haplotypes. In recent years the accuracy of long-read sequencing techniques, such as Oxford Nanopore Technologies (ONT) and Pacific Biosciences (PacBio), has significantly improved. Already, long-read sequencing can be used to reach an unprecedented genetic resolution (Garg, 2021), e.g. by covering previously inaccessible genomic regions or entire RNA transcripts in individual reads. Hence, long-read sequencing is a game changer in genetic research for many fields. Advancements in sequencing technology require major, simultaneous algorithmic developments, for example, in the context of base calling, variant calling and assembly.

The latest ONT Q20+ and PacBio high fidelity (HiFi) protocols have revolutionized sequencing by producing reads with lengths in the order of tens of kilobases up to megabases and with base accuracies exceeding 99% (Logsdon et al., 2020). While the HiFi technology produces relatively more accurate sequences than ONT, ONT can generate ultra-long reads and is scalable from portable devices to benchtop sequencers. The key requirement of both technologies is high molecular weight DNA.

Rapidly decreasing sequencing costs have fueled the generation of increasing amounts of genomic data for biodiversity applications and human health (De Coster et al., 2021; Miller et al., 2021). An example is the implementation of next-generation sequencing (NGS) into clinical practice for diagnosis, prognosis, and therapy selection in various medical fields, of which oncology has been a pioneer (Berger and Mardis, 2018). Undoubtedly, challenges still exist, such as input material requirements and establishing robust data analysis methods. Further, extensive method development and benchmarking are needed and ongoing to fully unlock the potential of long reads for these and other applications (Amarasinghe et al., 2020).

In this Research Topic, we cover a range of investigations that all benefit in different ways from long reads: Transcriptome profiling (Vogeley et al.), variant calling (specifically, structural variants (Bolognini and Magi) and somatic mitochondrial variants (Lüth et al.), detection of circular DNA (Tüns et al.) and metagenome assembly (Luo et al.). In the following, we outline the long-read sequencing context and contribution of these works.

The portability of ONT’s MinION long-read sequencing device has increased IT infrastructure challenges associated with integrating these sequencers into a diagnostic laboratory setting whilst increasing the flexibility where sequencing can occur, ultimately resulting in better sample access and rapid results for patients. Addressing the corresponding need for immediate data analysis, (Vogeley et al.) provide a self-contained transcriptome workflow for RNA sequencing data, which runs *via* a local webserver and explicitly supports long-read RNA-Seq. Since long reads span entire transcripts, isoform-level expression is more accurate than short read-based expression estimates (Chen et al., 2021). Accordingly, the workflow of (Vogeley et al.) quantifies expression, computes differential expression, performs pathway enrichment analyses, and generates visualizations such as expression heatmaps.

With respect to resolving genomic variation, particularly the confident detection of structural variation from NGS data remains a challenge with no single best practice approach. There is much to be gained from using long-read sequencing data for structural variant (SV) calling, as the data is free of technical limitations such as genome coverage bias and alignment uncertainty, which plague the detection of structural variation in short-read sequencing. However, SV detection from long-read sequencing data needs dedicated tools different from those used for detecting SVs from short-read data. Meanwhile, these recent tools still require comprehensive benchmarking. Accordingly, for both real and simulated ONT data, (Bolognini and Magi) compare five long read SV callers across four long read aligners and assess the effect of sequencing parameters on performance.

Another type of genetic alteration is somatic variation, i.e., mutations acquired during life course. Somatic variant calling in the context of human mitochondrial genetics has been investigated by (Lüth et al.). Mitochondria are maternally inherited cell organelles that carry a circular genome. Lüth et al. generated mixtures of two mitochondrial haplotypes at different ratios for benchmarking somatic variant calling from Nanopore sequencing data with respect to variant calls obtained from deep short-read sequencing. They compared two mappers and three callers and found that basecaller, mapper, and variant caller choices affect performance. Further, somatic variants at allele frequencies of 5% are largely accurately detected, but performance decreases significantly for lower frequencies.

Another Research Topic article investigates the detection of circular DNA from Nanopore sequencing data. Extra-chromosomal DNA (ecDNA) is common in cancer and plays a crucial role in tumor progression. (Tüns et al.) present a novel open-source workflow that processes Nanopore data for detecting circular ecDNA. Its key step is a dedicated graph-theoretic approach devised by the authors. They demonstrated the workflow for the MYCN oncogene and found ecDNA’s breakpoints reliably at the base level. The work of (Tüns et al.) provides readily and comprehensive detection of circular DNAs from long-read Nanopore sequencing, which facilitates biomarker discovery for cancer progression.

Finally, (Luo et al.) improve long-read sequencing-based metagenome assembly by performing read correction using two complementary correction tools and then merging corrected reads before assembly. While conceptually simple, this strategy considerably improves a range of assembly quality criteria compared to stand-alone state-of-the-art metagenome assemblers, both on simulated and real data. Consequently, their corresponding workflow MetaBooster generates assemblies down to the level of individual strains.

In summary, our Research Topic captures the current state of long-read sequencing: All investigations covered (RNA-Seq, SVs, somatic variants, metagenomics) clearly benefit from using long reads, some are even only possible using long-read sequencing (e.g., detection of circular DNAs). However, most applications still need benchmarking to validate them with respect to short-read sequencing (Lüth et al.) or to find a good selection or combination of analysis tools (Bolognini and Magi; Luo et al.; Lüth et al.). Finally, developments allowing rapid, automated analysis of long-read sequencing data are brought forward (Vogeley et al.), highlighting that broad implementation of tools and workflows into biomedical and clinical settings is close.
